# Editorial: Targeting glucose metabolism in cancer immunity and immunotherapy

**DOI:** 10.3389/fimmu.2023.1171274

**Published:** 2023-03-08

**Authors:** Olivier Feron, Chih-Hao Chang, Frédérique Végran

**Affiliations:** ^1^ Pole of Pharmacology and Therapeutics, Institut de Recherche Expérimentale et Clinique (IREC), UCLouvain, Brussels, Belgium; ^2^ WELBIO department, WEL Research Institute, Wavre, Belgium; ^3^ The Jackson Laboratory, Bar Harbor, ME, United States; ^4^ Université de Bourgogne Franche-Comté, Dijon, France; ^5^ CRI UMR 1231 Lipides, Nutrition and Cancer Team CAdIR, Dijon, France; ^6^ Centre Georges-François Leclerc, UNICANCER, Dijon, France; ^7^ LipSTIC Labex, Dijon, France

**Keywords:** glycolysis, immunometabolism, fluorodeoxyglucose, gamma-glutamyl hydrolase, GLUT3, glucose transporter, pancreatic cancer

Glucose catabolism through glycolysis, the TCA cycle and OXPHOS is a source of ATP and biosynthetic intermediates. In healthy cells, glucose metabolism is tightly regulated by insulin and growth factors to ensure that only the required amounts are taken up. By contrast, cancer cells often have oncogenic alterations in signaling pathways, such as PI3K/Akt activation, that enhance their ability to take up glucose from the extracellular environment. This phenomenon is known as the Warburg effect, whereby cancer cells ferment glucose into lactate, even in the presence of oxygen to support *de novo* generation of nucleotides, lipids, proteins and antioxidant defenses. It is important to note that glucose is not only essential for cancer cell metabolism, but also for immune cells in the tumor microenvironment. In fact, glucose competition between cancer and immune cells can have a profound impact on the ability of the latter to generate an efficient immune response against the tumor. For example, upon activation, T cells undergo metabolic reprogramming characterized by a shift from oxidative catabolic metabolism in naive T cells to anabolic glycolytic metabolism. This shift in metabolic preferences helps to meet the energy and biosynthetic demands required for T cell proliferation and effector functions. Glucose competition within the tumor microenvironment can therefore reduce the capacity of T cells to produce important cytokines such as IFN-γ, thereby diminishing their ability to mount an efficient immune response. Thus, understanding the complex interplay between glucose metabolism and the immune response in the tumor microenvironment is crucial for developing effective cancer immunotherapies.

In this collection, Cribioli et al. report that enforced expression of the glucose transporter GLUT3 supports the metabolic fitness of T cells and augments their anticancer activity. GLUT3 has both a higher affinity for glucose and increased transport capacity than GLUT1, and thus represents an attractive candidate for T cell bioengineering. Contrary to strategies based on drug exposure of T cells before adoptive transfer to enhance *in vivo* persistence, genetic engineering may offer T cells a permanent advantage unlikely to be lost in response to the immunosuppressive tumor microenvironment. The authors further indicate that enforcing expression of GLUT3 by T cells increases glucose uptake and importantly drives the accumulation of energy reserves in the form of glycogen and lipids. These metabolic alterations contribute to significantly improved anticancer immune response measured both *in vitro* and *in vivo*. Notably, this stimulated glycolytic turnover did not lead to T-cell exhaustion, strengthening the clinical potential of this approach.

Interestingly, nowadays the most widely used molecular imaging tracer is ^18^F-fluoro-2-deoxy-D-glucose (FDG) that is detected by positron emission tomography-computed tomography (PET/CT). Although FDG-PET is a non-invasive imaging method that has shown significant predictive and prognostic value for numerous cancer types, its reliance on a single metabolic parameter index has limitations. Combining FDG-PET with evaluation of biological parameters could be particularly useful in patients with advanced NSCLC, where information on both the tumor burden and inflammatory status offers a better predictive capacity of response to immune checkpoint inhibitors (ICI). In this collection, Jin et al. report that a combination of tumor and secondary lymphoid ^18^F-FDG PET/CT metabolic parameters better reflects the immune status of the human body and the anticipated response to immunotherapy. The study found that high total metabolic tumor volume (TMTV) associated with a high spleen-to-liver SUVmax ratio (SLR) was actually associated with poor prognosis. By contrast, increased splenic FDG uptake post-ICI treatment (ie, systemic immune activation) predicted survival benefits. These findings contribute to a more precise evaluation of the immune status and potential response to immunotherapy in patients with advanced NSCLC.

A study by Chen et al. focuses on using new molecular markers for early detection, diagnosis, and monitoring of cancers. Specifically, they investigated gamma-glutamyl hydrolase (GGH), a key enzyme in folate metabolism pathway. High expression of GGH is associated with resistance to antifolate drugs such as methotrexate and poor prognosis in general. Through transcriptomic analysis, these authors clustered colon cancer cases into four metabolic subtypes named quiescent, glycolytic, cholesterogenic, and mixed. These metabolic subtypes differ with respect to the immune score, for instance, with the cholesterogenic subtype (but not the glycolytic subtype) exhibiting an inflammatory subtype. A differentially expressed gene (DEG) study, further revealed a correlation between upregulated GGH in colon cancer and CD4^+^ T-cell infiltration, making it a favorable independent prognostic factor. Interestingly, GGH overexpression in colon cancer cell lines was associated with a reduced expression of glycolytic enzymes and on the contrary, GGH silencing led to an increased glycolytic turnover both *in vitro* and *in vivo*. These findings highlight the potential of GGH as a prognostic biomarker and suggest that targeting GGH may be a promising strategy for cancer treatment.

Finally, another article in this collection by Dong et al. delves into the role of glycolysis in pancreatic tumors, including cancer and non-cancer cells. The authors provide a comprehensive description of situations in which glucose uptake and availability drive phenotypic alterations, highlighting various areas where modulating the glycolytic pathway could be beneficial in reducing or preventing drug resistance and immune tolerance.

This special edition Research Topic puts a spotlight on the ongoing progresses in the growing field of immunometabolism. The studies listed above may provide valuable guidance and direction to researchers in this field.

## Author contributions

OF wrote this editorial, FV and C-HC contributed to manuscript revision. All the authors approved the submitted version.

